# Propionate and butyrate attenuate macrophage pyroptosis and osteoclastogenesis induced by CoCrMo alloy particles

**DOI:** 10.1186/s40779-022-00404-0

**Published:** 2022-08-23

**Authors:** Yang-Lin Wu, Chen-Hui Zhang, Yun Teng, Ying Pan, Nai-Cheng Liu, Pei-Xin Liu, Xu Zhu, Xin-Lin Su, Jun Lin

**Affiliations:** 1 Department of Orthopaedics, Suzhou Dushu Lake Hospital, Dushu Lake Hospital Affiliated to Soochow University, Medical Centre of Soochow University, Suzhou, 215001 Jiangsu China; 2 Department of Orthopaedics, The First Affiliated Hospital of Soochow University, Soochow University, Suzhou, 215006 Jiangsu China; 3 Department of Infectious Diseases, The Second Affiliated Hospital, Zhejiang University School of Medicine, Hangzhou, 310058 China

**Keywords:** NLRP3 inflammasome, Pyroptosis, Short chain fatty acids, Osteolysis, Osteoclast

## Abstract

**Background:**

Wear particles-induced osteolysis is a major long-term complication after total joint arthroplasty. Up to now, there is no effective treatment for wear particles-induced osteolysis except for the revision surgery, which is a heavy psychological and economic burden to patients. A metabolite of gut microbiota, short chain fatty acids (SCFAs), has been reported to be beneficial for many chronic inflammatory diseases. This study aimed to investigate the therapeutic effect of SCFAs on osteolysis.

**Methods:**

A model of inflammatory osteolysis was established by applying CoCrMo alloy particles to mouse calvarium. After two weeks of intervention, the anti-inflammatory effects of SCFAs on wear particle-induced osteolysis were evaluated by Micro-CT analysis and immunohistochemistry staining. In vitro study, lipopolysaccharide (LPS) primed bone marrow-derived macrophages (BMDMs) and Tohoku Hospital Pediatrics-1 (THP-1) macrophages were stimulated with CoCrMo particles to activate inflammasome in the presence of acetate (C2), propionate (C3), and butyrate (C4). Western blotting, Enzyme-linked immunosorbent assay, and immunofluorescence were used to detect the activation of NLRP3 inflammasome. The effects of SCFAs on osteoclasts were evaluate by qRT-PCR, Western blotting, immunofluorescence, and tartrate-resistant acid phosphatase (TRAP) staining. Additionally, histone deacetylase (HDAC) inhibitors, agonists of GPR41, GPR43, and GPR109A were applied to confirm the underlying mechanism of SCFAs on the inflammasome activation of macrophages and osteoclastogenesis.

**Results:**

C3 and C4 but not C2 could alleviate wear particles-induced osteolysis with fewer bone erosion pits (*P* < 0.001), higher level of bone volume to tissue volume (BV/TV, *P* < 0.001), bone mineral density (BMD, *P* < 0.001), and a lower total porosity (*P* < 0.001). C3 and C4 prevented CoCrMo alloy particles-induced ASC speck formation and nucleation-induced oligomerization, suppressing the cleavage of caspase-1 (*P* < 0.05) and IL-1β (*P* < 0.05) stimulated by CoCrMo alloy particles. C3 and C4 also inhibited the generation of Gasdermin D-N-terminal fragment (GSDMD-NT) to regulate pyroptosis. Besides, C3 and C4 have a negative impact on osteoclast differentiation (*P* < 0.05) and its function (*P* < 0.05), affecting the podosome arrangement and morphologically normal podosome belts formation.

**Conclusion:**

Our work showed that C3 and C4 are qualified candidates for the treatment of wear particle-induced osteolysis.

**Supplementary Information:**

The online version contains supplementary material available at 10.1186/s40779-022-00404-0.

## Background

As one of the most advanced treatments for arthritis, more than one million total joint replacement (TJR) surgeries are performed every year [1]. Long-term studies show that nearly 20% of patients will gradually develop periprosthetic osteolysis and bone loss, which means revision surgery is inevitable. If left untreated, periprosthetic osteolysis will eventually cause joint loosening and even bone loss [2]. The whole pathological process is closely related to the wear particles produced by the implant. These wear particles can be phagocytosed by surrounding macrophages [3]. After uptake of small wear-particles, these myeloid immune cells will be stimulated to secrete inflammatory cytokines including interleukin 1 beta (IL-1β), interleukin 6 (IL-6), prostaglandin E2 (PGE2), and tumour necrosis factor (TNF). These cytokines, especially IL-1β, are associated with osteoclast activation and bone resorption [4–6].

The NLRP3 inflammasome consists of a sensor (Nod-like receptor pyrin domain 3, Nlrp3), an adaptor (apoptosis-associated speck-like protein containing a caspase recruitment domain, ASC), and an effector (caspase-1). Inflammasomes are large multiprotein platforms that sense a variety of microbial, environmental, and endogenous stressors leading to the maturation of IL-1β and IL-18 [7–9]. ASC will be recruited by inflammasome sensors upon activation. Then, multiple ASC filaments are combined into a macromolecular focus called the ASC speck. Assembled ASC recruits caspase-1 and enables caspase-1 cleavage and activation. The active caspase-1 is sufficient for gasdermin D (GSDMD) to generate GSDMD-N-terminal fragments (GSDMD-NT), which in turn form pores in the membrane to regulate IL-1β release, known as pyroptosis [10–15].

Particulates including asbestos [16], silica [17], monosodium urate (MSU) crystals [18, 19], wear particles [20], and cholesterol crystals [21–23] have been reported to be sufficient for NLRP3 inflammasome activation. As a key step, NLRP3 inflammasome activation in macrophages upon wear particle stimulation would be followed by implant loosening and periprosthetic osteolysis after TJR [24–27]. Our previous work revealed that C4, one type of short chain fatty acids (SCFAs), inhibited NLRP3 inflammasome activation induced by titanium particles and alleviated osteolysis [28]. As products of the microbial fermentative activity in the distal small intestine and colon, SCFAs mainly consist of acetate (C2), propionate (C3), and butyrate (C4) [29–31]. Several studies highlighted the bone metabolism-regulating and immunomodulatory capacities of SCFAs [32–36]. Recently, Yuan et al. [37] reported the differential effect of SCFAs on endothelial cell inflammasome activation. Hence, we hypothesised that SCFAs may also exert different effects on CoCrMo alloy particle-induced NLRP3 inflammasome activation.

Up to now, the underlying mechanism that leads to NLRP3 inflammasome activation by wear particles remains incompletely characterized. We aimed to determine how wear particles activated the NLRP3 inflammasome of macrophages and subsequent pyroptosis. In this study, we also explored the differential effect of SCFAs on inflammasome activation and pyroptosis induced by CoCrMo alloy particles. Furthermore, we investigated the effect of SCFAs on osteoclastogenesis promoted by IL-1β.

## Methods

### Animal study

C57BL/6J male mice, 8 – 10 weeks old, were bought from Suzhou Healthytech Bio-pharmaceutical Co., Ltd. (Suzhou, China). All mice (*n* = 60) were maintained in a specific-pathogen-free (SPF) environment with free access to water and chow under the standard 12 h-light/12 h-dark cycle at 22 – 24.5 °C. After acclimatizing for two weeks, mice were randomly assigned to the following groups to evaluate the effect of SCFA treatment: sham (*n* = 12), CoCrMo (*n* = 12), C2 (*n* = 12), C3 (*n* = 12), and C4 (*n* = 12) groups.

All mice were then subjected to osteolysis surgery as previously described [38, 39]. Briefly, CoCrMo alloy particles were incubated at 180 °C for 8 h to remove endotoxins then transferred to ethanol solution for 1 d. Then, CoCrMo alloy particles were resuspended in sterile PBS at a final concentration of 500 mg/ml for subsequent studies. A 10 mm midline sagittal incision was created over the calvarium of each mouse and 40 μl of CoCrMo alloy particles at a concentration of 500 mg/ml were administered. Mice in the Sham group received sham operation without treatment. All animal studies were approved by the Dushu Lake hospital affiliated to Soochow University. The research was conducted according to the principles and procedures of the ethics committee of the Dushu Lake Hospital Affiliated to Soochow University.

After surgery, mice in the C2, C3, and C4 groups received water with 150 mmol/L sodium acetate, propionate, and butyrate (Sigma-Aldrich St. Louis, MO, USA), respectively. Sham and CoCrMo groups were supplied with sterile water matched for sodium content and pH.

### Micro-CT analysis

The calvaria collected from each group were examined by micro-computed tomography (micro-CT) scanning (SkyScan 1176, Aartselaar, Belgium). The parameters of the X-ray were set at a current of 500 µA with a voltage of 50 kV, 9 µm per layer. For quantification, a CT analyser (SkyScan) was used. A circular (3-mm diameter) region of interest (ROI) was selected with ten layers on each mouse calvaria specimen. Then, 3D reconstruction and histomorphometric measurements of bone mineral density (BMD, mg/cc), the ratio of bone volume to tissue volume (BV/TV, %), and total porosity (%) were performed by the CT analyser (SkyScan).

### Cell culture and stimulation

#### Differentiation of bone marrow-derived macrophages (BMDMs)

Bilateral femora and tibiae of mice from wild type (WT) and G protein-coupled receptor 109a knockout (GPR109A^−/−^) mice (*n* = 5) were isolated to obtain bone marrow-derived macrophages (BMDMs). Both ends of the bones were cut, and the bone marrow was flushed out with Dulbecco’s modified Eagle’s medium (DMEM). After red blood cell lysis, the bone marrow cells were resuspended at (2 – 4) × 10^6^ cells/ml in DMEM supplemented with 10% FBS and 50 ng/ml M-CSF (R&D Systems, Minneapolis, MN, USA) and then placed into 6-well plates. The purity of BMDMs was assessed by flow cytometry using CD11b and F4/80 antibodies and was routinely > 94.5% (Additional file 1: Fig. S1), which is similar to a previous study [40]. The cells were used for further studies after 5–7 d of culture. To activate the inflammasome in BMDMs, the cells were primed with 100 ng/ml lipopolysaccharide (LPS) for 3 h and then stimulated with CoCrMo alloy particles (0.1 mg/ml) for 6 h in the presence of acetate, propionate, butyrate, trichostatin A (TSA), Panobinostat, AR420626, 4-CMTB, and niacin.

#### THP-1 macrophages

THP-1 cells were brought from Procell Life Science&Technology Co., Ltd. (Procell, Wuhan, China) and cultured in RPMI 1640 medium (Procell, Wuhan, China) with 10% foetal bovine serum and 1% antibiotic solution. THP-1 cells were differentiated to macrophages by 3 h incubation with 100 nmol/L phorbol-12-myristate-13-acetate (PMA) (MedChemExpress, Monmouth Junction, NJ, USA). Then THP-1 macrophages were primed with 100 ng/ml LPS (Sigma) for 3 h and stimulated with CoCrMo alloy particles with different doses of acetate, propionate, and butyrate.

#### Osteoclast differentiation

Total bone marrow cells from WT and GPR109A^−/−^ mice were isolated by flushing the femora and tibiae. The cells were plated overnight at 37 °C with 5.5% CO_2_ in alpha minimum essential medium (αMEM), supplemented with 10% FBS and 1% penicillin/streptomycin (Gibco, Carlsbad, CA, USA), and 40 ng/ml macrophage colony stimulating factor (M-CSF; R&D Systems). Next day, nonadherent cells were collected and cultured in osteoclast medium with 40 ng/ml M-CSF and 50 ng/ml receptor activator of nuclear factor kappa-B (NF-κB) ligand (RANKL; R&D Systems) in 24-well plates (for tartrate-resistant acid phosphatase (TRAP) staining, immunofluorescence), 12-well plates (for RNA isolation), or 6-well plates (for Western blotting) at a density of 1 × 10^6^ cells/ml at 37 °C and 5.5% CO_2_. The medium was changed every 3 d. In SCFA experiments, preosteoclasts in C3 and C4 groups were further stimulated on day 1 of culture with the following stimulants: 5 mmol/L propionate, 1 mmol/L butyrate (Sigma-Aldrich), 40 ng/ml IL-1β (MedChemExpress), 50 nmol/L TSA (MedChemExpress), 25 nmol/L Panobinostat (Sigma-Aldrich), 100 μmol/L 4-CMTB (Sigma-Aldrich), 1 mmol/L niacin (MedChemExpress), or 25 μmol/L AR42062 (Sigma-Aldrich). Then, fully differentiated osteoclasts (days 5–7) were collected for PCR, Western blotting, TRAP staining, and immunofluorescence staining.

### Enzyme-linked immunosorbent assay

IL-1β and IL-18 in cell supernatants were measured using ELISA kits (MultiSciences, Hangzhou, China) following the manufacturer’s instructions.

### Western blotting analysis

Cells were lysed in radio immunoprecipitation assay (RIPA) lysis buffer (Beyotime Institute of Biotechnology, Shanghai, China) containing proteinase and phosphatase inhibitors. A BCA assay kit (Beyotime) was used to measure the concentration of protein. The supernatant proteins were precipitated. The protein of lytic samples was transferred to polyvinylidene fluoride (PVDF) membranes (Beyotime, China) after separating by sodium dodecyl sulfate polyacrylamide gel electrophoresis (SDS-PAGE) (Beyotime). Membranes were incubated with antibodies against gasdermin D (GSDMD, Abcam, Cambridge, UK), IL-1β (Abcam), nuclear factor of activated T-cells, cytoplasmic 1 (NFATc-1, Abcam), TRAP (Abcam), pro caspase-1 (Cell Signaling Technology, Danvers, MA, USA), cleaved caspase-1 (Cell Signaling Technology), ASC (Cell Signaling Technology), TNF receptor-associated factor (TRAF) 2 (Proteintech, Rosemont, IL, USA), TRAF6 (Proteintech), c-Fos (Proteintech), cathepsin K (CTSK, Proteintech), matrix metalloprotein 9 (MMP9, Proteintech), and anti-actin (Beyotime) overnight at 4 °C after blocking with quick block buffer (Beyotime) for 30 min. Then, membranes were washed three times with TBS-Tween and incubated with the appropriate secondary antibody for 1 h at room temperature. The results were visualised via chemiluminescent peroxidase substrate (Proteintech).

### Quantitative real-time polymerase chain reaction (qRT-PCR)

Real-time RT-PCR was performed to assess the mRNA levels of *NFATc-1*, osteoclast stimulatory transmembrane protein (*Ocstamp*), osteoclast-associated immunoglobulin-like receptor (*Oscar*), *Trap*, carbonic anhydrase 2 (*Car2*), *Ctsk*, and *Mmp9*. Total RNA was extracted using TRIzol® reagent according to the manufacturer's instructions. Complementary DNA (cDNA) from each sample was reverse transcribed from 1 μg RNA in each group using a RevertAid First Strand cDNA Synthesis Kit (Thermo Fisher Scientific, Waltham, MA, USA). Subsequently, cDNA was amplified through quantitative PCR using the iTaq™ Universal SYBR® Green Supermix kit (Bio Rad, Hercules, CA, USA). The primer sequences are listed in Additional file 2: Table S1.

### Histology, immunohistochemistry and immunofluorescence staining

Each mouse calvarium was embedded in paraffin, then cut into 6 µm slices. Haematoxylin and eosin (HE) staining was performed according to the manufacturer’s instructions. TRAP staining was conducted using a TRAP staining kit (BZ Biotechnology, Suzhou, China) to detect osteoclasts.

For immunohistochemistry, 6 µm sections of calvaria were cut and subjected to heat-induced antigen retrieval. Then the calvarial sections were incubated with anti-IL-1β (rabbit, Cell Signaling Technology), anti-NFATc-1 (rabbit, Abcam), anti-CTSK, and anti-MMP9 (Mouse, Santa Cruz Biotechnology, Santa Cruz, CA, USA) primary antibodies overnight at 4 °C. Next day, the sections were incubated with the appropriate secondary antibody for 1 h at room temperature then stained for 2–6 min with 100 μl 3,3′-diaminobenzidine (DAB).

Immunofluorescence in osteoclasts. For immunofluorescence, 1 × 10^6^ cells/well were cultured in 1 ml/well osteoclast medium with supplements on 12-mm diameter coverslips (Biosharp, Hefei, China) in 24-well plates at 37 °C and 5.5% CO_2_ until fully differentiated. After fixation, the cells were stained with Acti-stain 670 phalloidin (Beyotime) according to the manufacturer’s instructions to visualise F-actin ring formation. Cells were also stained with antibodies against TRAF2, NFATc-1, and 2-(4-amidinophenyl)-6-indolecarbamidine dihydrochloride (DAPI) for osteoclast immunofluorescence.

### Statistical analysis

Statistical analysis was performed by a one-way analysis of variance (ANOVA) and post-hoc (Tukey) test within Sigmaplot 12.5 software (Systat Software, San Jose, CA, USA). Data are presented as mean ± SEM. *P* < 0.05 was considered statistically significant.

## Results

### C3 and C4 alleviate osteolysis in vivo

**Fig. 1:**
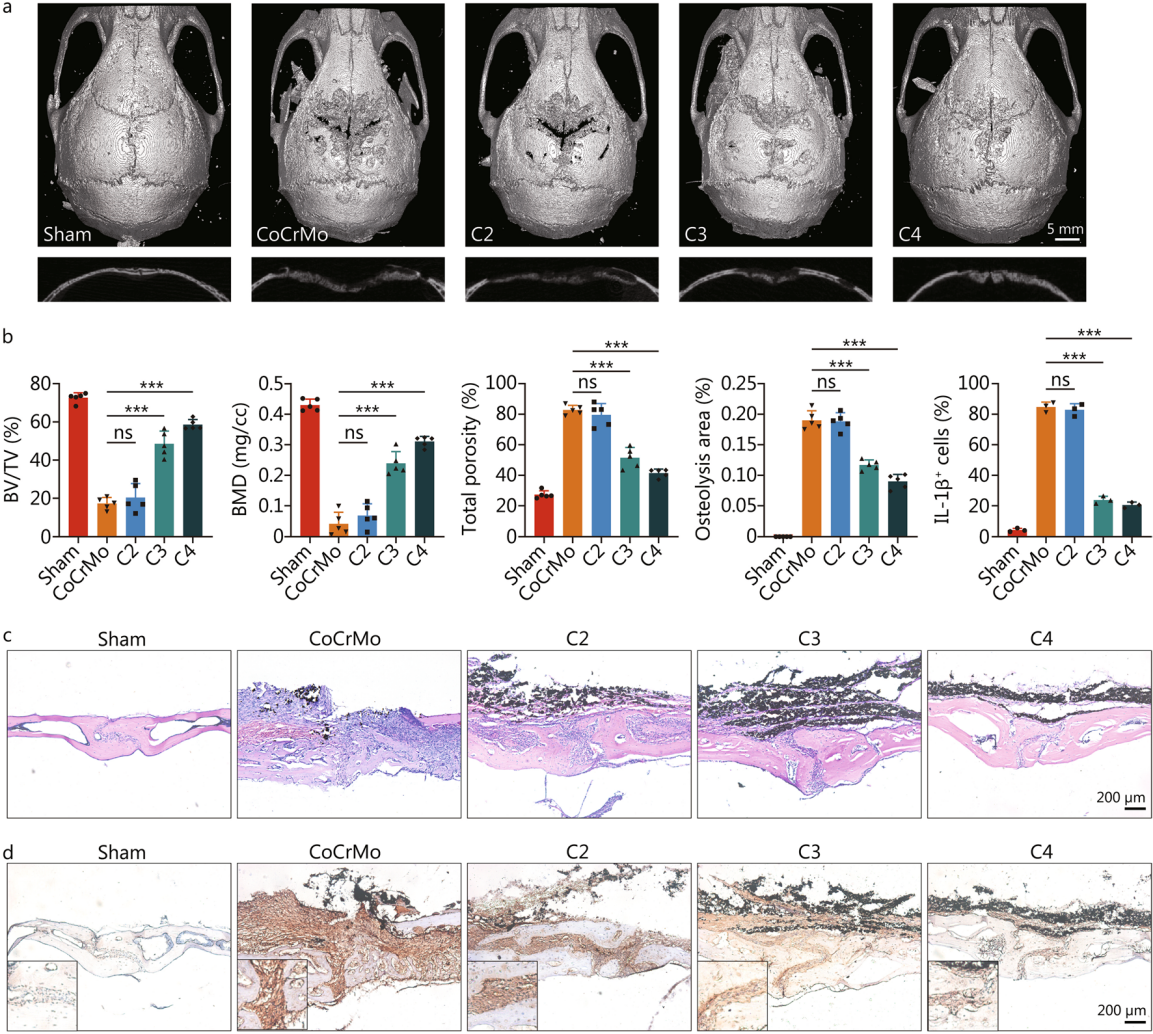
C3 and C4 alleviate osteolysis in vivo. **a** Representative 3D reconstruction (micro-CT) image of calvarium in each group. **b** Quantification of BV/TV (bone volume to tissue volume), BMD (bone mineral density), total porosity, osteolysis area (*n* = 5), and IL-1β-positive cells (*n* = 3). **c** Representative H&E staining of calvarial slices from each group. **d** Representative staining of IL-1β immunohistochemistry from each group. Results are mean ± SEM, ^***^P < 0.001, ns non-significant. C2 acetate, C3 propionate, C4 butyrate, BV/TV bone volume to tissue volume, BMD bone mineral density

Given that NLRP3 inflammasome activation by wear particles is a key step in the course of osteolysis [24–27], a wear particle-induced osteolysis mouse model was used for in vivo studies. We found that only C3 and C4 had a therapeutic effect on osteolysis. As micro-CT 3D reconstruction images showed, osteolysis caused by CoCrMo alloy particles resulted in a large amount of bone loss, while only a few erosion pits (osteolysis area, *P* < 0.001) were observed in the calvarium following treatment with C3 or C4, with a higher level of BV/TV (*P* < 0.001), BMD (*P* < 0.001), and a lower total porosity (*P* < 0.001; Fig. 1a, b). HE staining and immunohistochemical staining of IL-1β also showed the anti-inflammatory effect of C3 and C4 with fewer IL-1β-positive cells (*P* < 0.001; Fig. 1b-d). However, these beneficial effects on osteolysis were not observed in the mice treated with C2. Thus, our work showed that different SCFAs also have differential effects on wear particle-induced osteolysis.

### C3 and C4 inhibit activation of the NLRP3 inflammasome stimulated by CoCrMo alloy particles

**Fig. 2:**
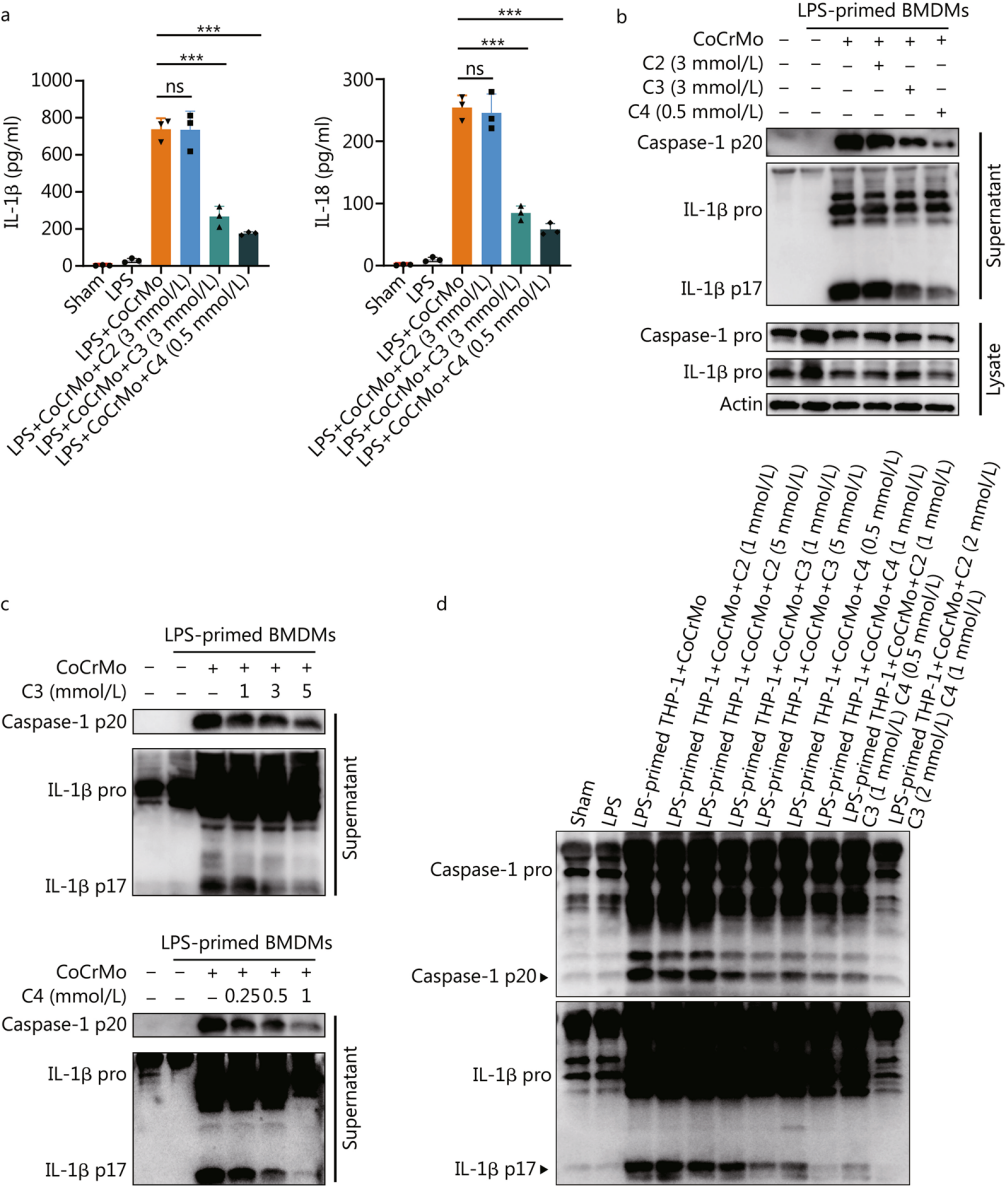
C3 and C4 inhibit NLRP3 inflammasome activation by CoCrMo alloy particles. BMDMs (LPS-primed) were treated with C3 or C4 and then stimulated with CoCrMo alloy particles. **a** Supernatants were collected for measuring IL-1β and IL-18 release by ELISA. **b, c** BMDMs (LPS-primed) were treated with different doses of C2, C3, C4, and then stimulated with CoCrMo alloy particles. Cell lysates and supernatants were analysed by immunoblotting. **d** LPS-primed THP-1 macrophages (PMA-differentiated) treated with C2, C3, C4, and then stimulated with CoCrMo alloy particles. Supernatants were analysed by immunoblotting. Results are mean ± SEM, ^***^P < 0.001, ns non-significant. BMDMs bone marrow-derived macrophages, C2 acetate, C3 propionate, C4 butyrate, IL-1β interleukin 1 beta, IL-18 interleukin 18, LPS lipopolysaccharide

We first investigated the effect of three different SCFAs on NLRP3 inflammasome activation of macrophages. After incubation with CoCrMo alloy particles, C3 and C4, but not C2, significantly blocked the release of IL-1β (*P* < 0.001) and IL-18 (*P* < 0.001) in LPS-primed BMDMs (Fig. 2a). Western blotting also confirmed that C3 and C4 effectively suppressed the cleavage of caspase-1 (*P* < 0.001) and IL-1β (*P* < 0.001, Fig. 2b and Additional file 1: Fig. S2a). Of note, the inhibitory effects of C3 and C4 were dose-dependent according to the results of immunoblotting (Fig. 2c and Additional file 1: Fig. S2b, c). Next, we applied a human macrophage cell line (PMA-differentiated THP-1 macrophages) to confirm that the suppression could also be observed in human macrophages. As we expected, only C3 and C4 significantly inhibited NLRP3 inflammasome activation by CoCrMo alloy particles (Fig. 2d and Additional file 1: Fig. S2d). Taken together, these data show that C3 and C4 restrained inflammasome activation and IL-1β release in macrophages.

### C3 and C4 inhibit ASC assembly and subsequent pyroptosis

**Fig. 3:**
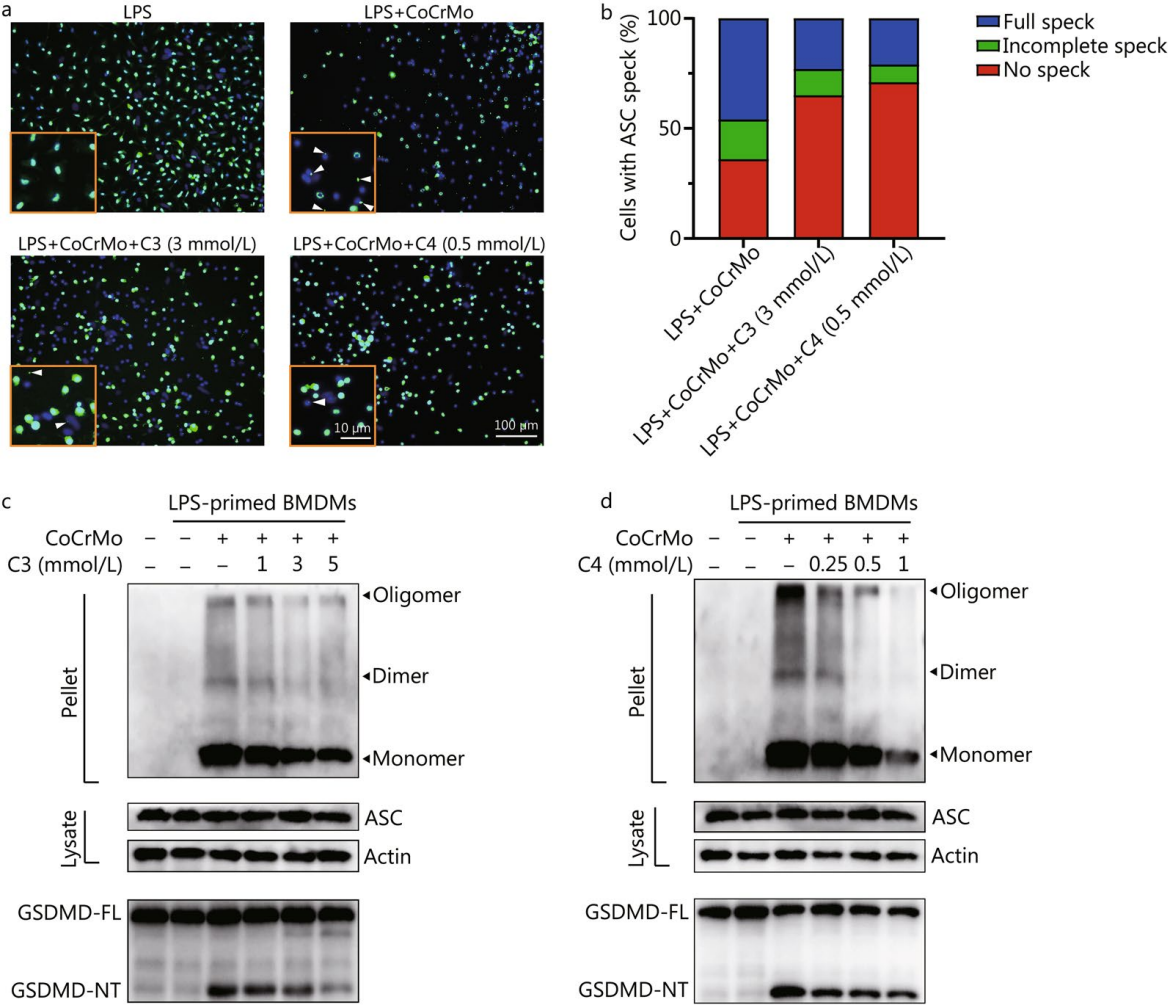
C3 and C4 block the assembly of ASC and pyroptosis stimulated by CoCrMo alloy particles in macrophages. **a** Representative immunofluorescence images of ASC speck formation in BMDMs (LPS-primed) stimulated with CoCrMo alloy particles in the presence of C3 or C4. **b** Quantification of ASC speck formation. BMDMs (LPS-primed) were treated with C3 **(c)** or C4 **(d)** and then stimulated with CoCrMo alloy particles. ASC oligomerization in cross-linked cytosolic pellets was analysed and GSDMD was detected by immunoblotting. ASC apoptosis-associated speck-like protein containing a caspase recruitment domain, GSDMD gasdermin D, GSDMD-NT gasdermin D-N-terminal fragment, C3 propionate, C4 butyrate, LPS lipopolysaccharide

To investigate how SCFAs inhibited NLRP3 inflammasome activation, we explored the effects of C3 and C4 on ASC nucleation-induced oligomerization or polymerization, which was considered to be a common mechanism of inflammasome activation. The formation of large ASC specks that polymerized and assembled by ASC upon stimulation with CoCrMo alloy particles was significantly reduced after intervention with C3 or C4 (Fig. 3a, b). Additionally, as the same effects on caspase-1 activity, C3 and C4 dose-dependently suppressed ASC oligomerization (Fig. 3c, d). Thus, C3 and C4 blocked NLRP3 inflammasome activation by preventing CoCrMo alloy particle-induced ASC oligomerization, speck formation, and assembly in BMDMs.

As a type of inflammatory cell death, pyroptosis is mediated by inflammasome activation. We speculated that inhibition of IL-1β secretion by C3 or C4 was attributed to pyroptosis. Then, GSDMD and its NT fragment, an effector protein of pyroptosis, were detected in the cell lysate by Western blotting. As we hypothesized, the GSDMD-NT, cleaved by active caspase-1 from GSDMD, was inhibited in a dose-dependent manner (Fig. 3c, d). The result of PI staining analysed by flow cytometry also revealed that cell death was reduced in the C3 and C4 treatment groups (Additional file 1: Fig. S3). Furthermore, the immunofluorescence of calcein/PI staining and lactate dehydrogenase (LDH) release assay showed that pyroptosis was suppressed after C3 or C4 treatment (*P* < 0.01, Additional file 1: Fig. S4). Thus, the inhibitory effects of C3 and C4 on IL-1β secretion also relied on the restriction of GSDMD cleavage, a key protein in pyroptosis.

### Effects of C3 do not depend on G protein-coupled receptors or HDAC inhibitor, while C4 requires GPR109A receptor

**Fig. 4:**
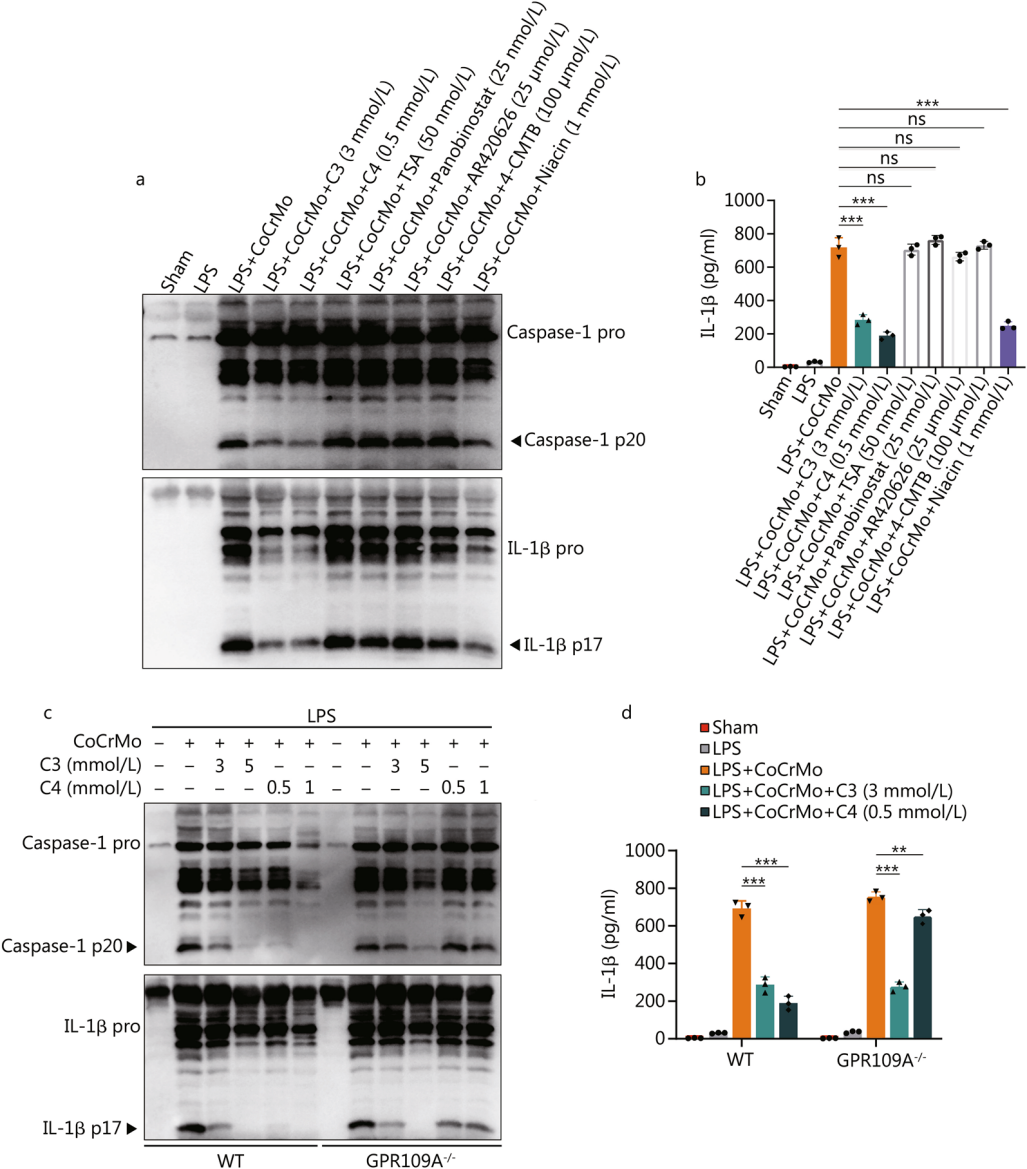
C3 inhibits NLRP3 inflammasome activation independently of GPCRs and HDAC inhibitor, while C4 is dependent on the GPR109A receptor. BMDMs (LPS-primed) treated with propionate (C3, 3 mmol/L), butyrate (C4, 0.5 mmol/L), TSA (50 nmol/L), Panobinostat (25 nmol/L), AR42062 (25 μmol/L), 4-CMTB (100 μmol/L), niacin (1 mmol/L) and then stimulated with CoCrMo alloy particles. Supernatants were analysed by immunoblotting for caspase-1, IL-1β activation **(a)**, and assayed for IL-1β secretion **(b)**. BMDMs (LPS-primed) from wild type (WT) and GPR109A^−/−^ mice treated with C3 or C4 and then stimulated with CoCrMo alloy particles. Supernatants were analysed by immunoblotting for caspase-1, IL-1β activation **(c)**, and assayed for IL-1β secretion **(d)**. Results are mean ± SEM, ^**^P < 0.01,^***^P < 0.001, ns non-significant. C3 propionate, C4 butyrate, IL-1β interleukin 1 beta, LPS lipopolysaccharide, TSA trichostatin A

C3 and C4 are known as signal molecules that can bind and activate G protein-coupled receptors (GPCRs) or act as a histone deacetylase (HDAC) inhibitor [41, 42]. We observed that neither TSA nor another HDAC inhibitor, Panobinostat, inhibited inflammasome activation or IL-1β secretion (Fig. 4a, b and Additional file 1: Fig. S5a). It has been suggested that C3 could activate GPR41 and partially GPR43, while C4 mainly binds and activates GPR109A and partially GPR41 [41]. Activation of GPR41 with the agonist AR420626, or of GPR43 with the agonist 4-CMTB did not affect inflammasome activation or IL-1β release (Fig. 4a, b and Additional file 1: Fig. S5a). However, niacin, a GPR109A agonist, had a similar inhibitory effect on CoCrMo alloy particle-induced inflammasome activation (*P* < 0.001; Fig. 4a, b and Additional file 1: Fig. S5a). This suggested that suppression of the inflammasome may be attributed to activation of the GPR109A receptor. Although we found that niacin did not have a powerful inhibitory effect on inflammasome activation at a concentration of 100 μmol/L in another study, the results of GPR109A-deficient BMDMs also revealed the important role of the GPR109A receptor in the benefits of C4. Compared with C4 in GPR109A-sufficient BMDMs, its inhibitory effect of C4 on CoCrMo alloy particle-induced inflammasome activation was greatly diminished in GPR109A-deficient BMDMs (Fig. 4c, d and Additional file 1: Fig. S5b). However, suppression of the inflammasome with C3 was not altered in GPR109A-deficient BMDMs. These results revealed that C3 did not depend on GPCRs or HDAC inhibition, while C4 required the GPR109A receptor to suppress the inflammasome activation induced by CoCrMo alloy particles.

### C3 and C4 inhibited osteoclast differentiation promoted by IL-1β

**Fig. 5:**
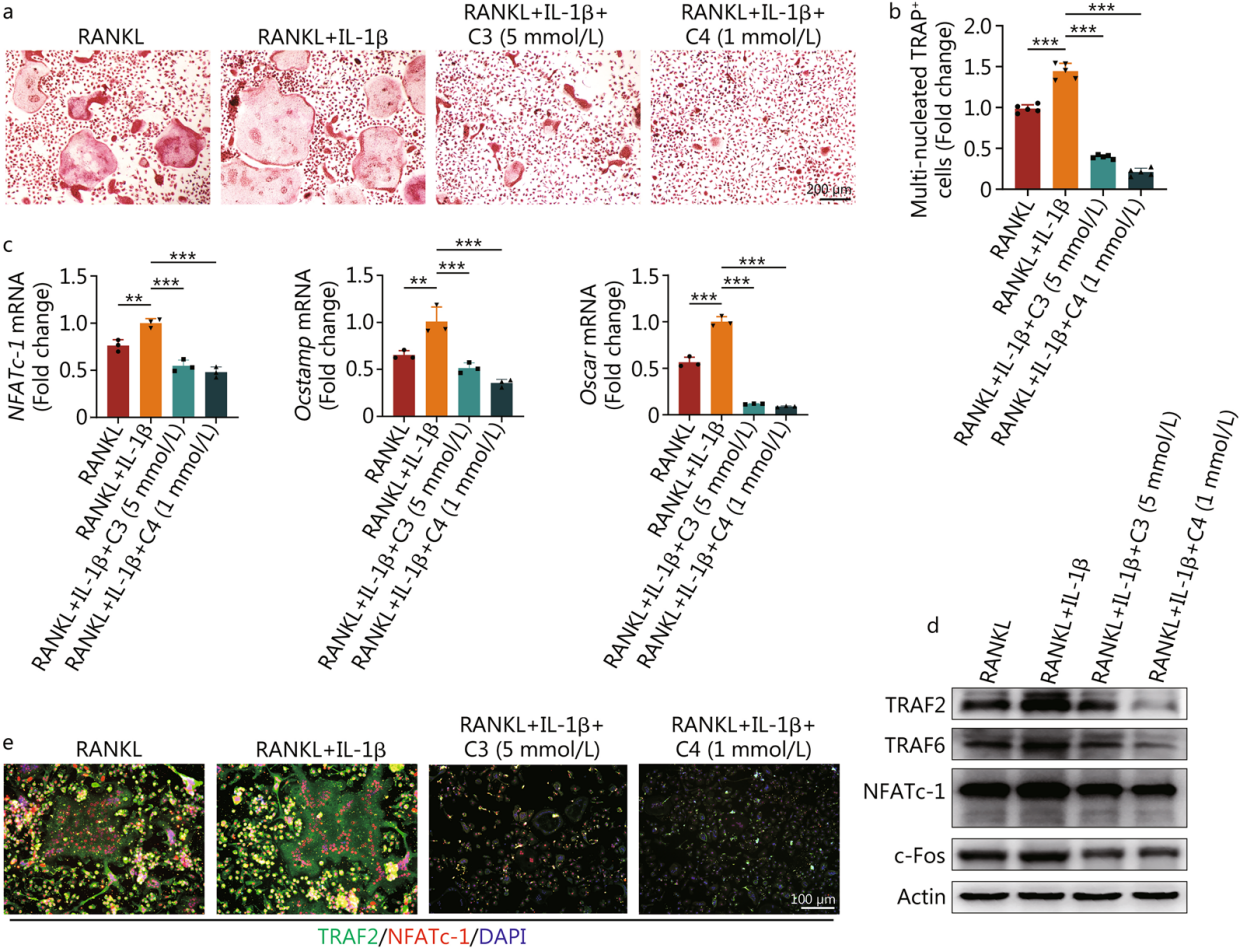
C3 and C4 inhibit osteoclast differentiation and formation stimulated by IL-1β. Pre-osteoclasts were cultured in the presence of C3 (5 mmol/L) or C4 (1 mmol/L) with IL-1β (40 ng/ml) for 5 d. Mature osteoclasts were harvested for TRAP staining (**a**) and quantification of TRAP-positive multi-nucleated (≥ 5 nuclei) osteoclasts (**b**). Relative expression of mRNA (**c**) and proteins in osteoclast differentiation (**d**) were analysed. **e** Representative images of osteoclast immunofluorescence co-immunostaining with TRAF2 (green), NFATc-1 (red), and DAPI (blue). Results are mean ± SEM, ^**^P < 0.01, ^***^P < 0.001. C3 propionate, C4 butyrate, IL-1β interleukin 1 beta, RANKL receptor activator of NF-κB ligand, TRAF2 TNF receptor-associated factor 2, NFATc-1 nuclear factor of activated T-cells

It has been suggested that inflammatory cytokines, especially IL-1β, could promote osteoclast differentiation and maturation [43]. Thus, we also investigated the effects of C3 and C4 treatment during osteoclastogenesis with IL-1β incubation. As previously reported, IL-1β greatly promoted the formation of osteoclasts. However, both C3 and C4 treatments significantly inhibited osteoclast formation (*P* < 0.001; Fig. 5a, b). To determine the underlying mechanism, we measured the transcript levels of genes expressed in the course of osteoclast differentiation. The diminished osteoclast numbers can be explained by reductions in the mRNA expression of *NFATc-1* (*P* < 0.001), osteoclast-stimulatory transmembrane protein (*Ocstamp*, *P* < 0.001), and osteoclast-associated immunoglobulin-like receptor (*Oscar*, *P* < 0.001, Fig. 5c). Furthermore, osteoclast differentiation-related proteins including TRAF2, TRAF6, NFATc-1, and c-Fos were promoted by IL-1β, while their expression was even lower than RANKL stimulation alone after C3 or C4 treatment (Fig. 5d and Additional file 1: Fig. S6). Double immunofluorescence of TRAF2 and NFATc-1 also confirmed the suppressive effects of C3 and C4 during osteoclast differentiation (Fig. 5e). Therefore, C3 and C4 not only inhibited NLRP3 inflammasome activation but also affected the osteoclast differentiation promoted by IL-1β.

### Osteoclasts treated with C3 or C4 showed lower lytic activity

**Fig. 6:**
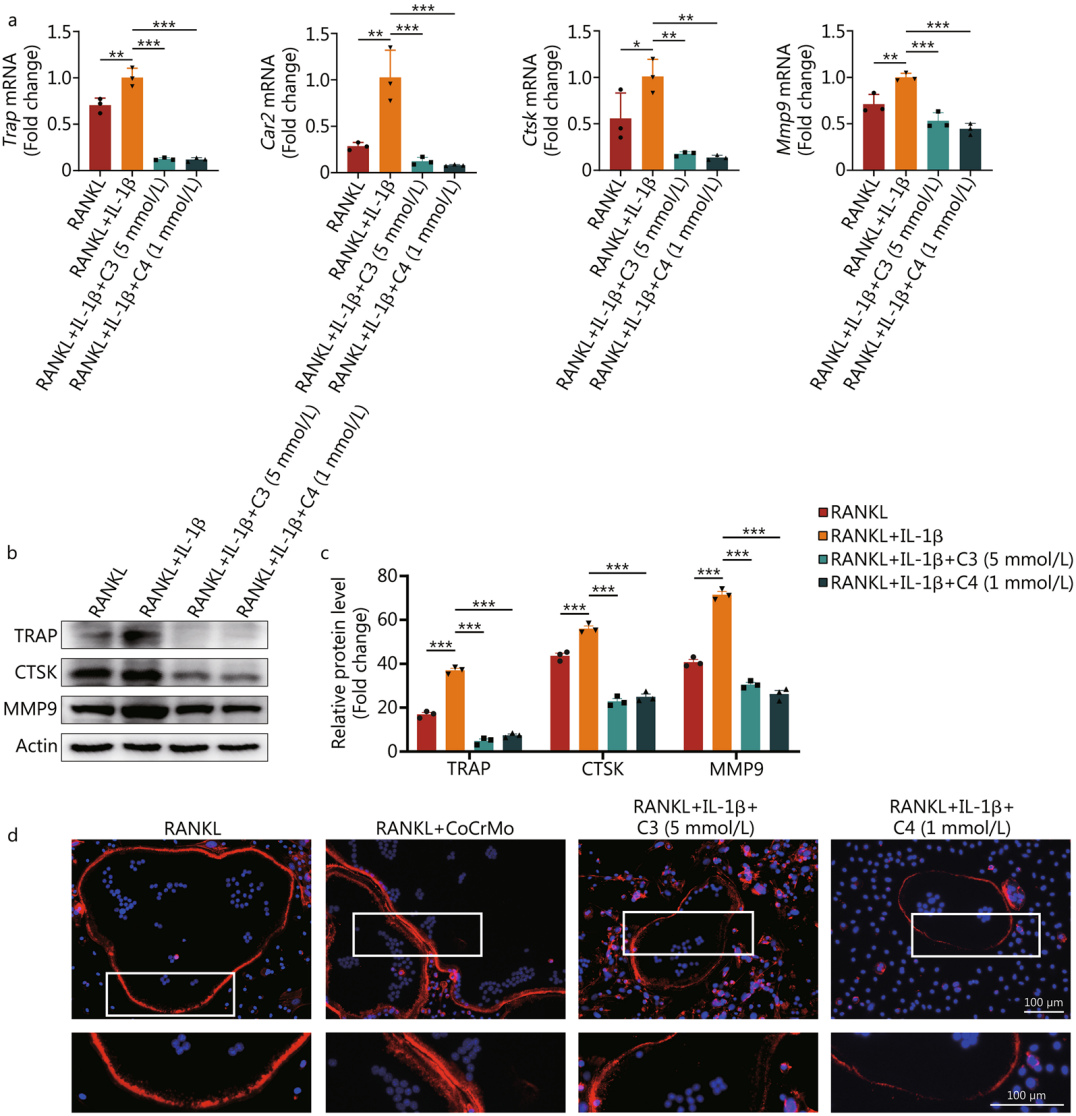
C3 and C4 diminish the lytic activity of osteoclasts. Pre-osteoclasts were cultured in the presence of C3 (5 mmol/L) or C4 (1 mmol/L) with IL-1β (40 ng/ml) for 5 d. Relative expression levels of bone resorption-related mRNA (**a**) and proteins (**b**) by osteoclasts. **c** Quantification of osteoclast bone resorption-related proteins. **d** Phalloidin labelling of F-actin in osteoclasts cultured on glass coverslips. Results are mean ± SEM, ^*^P < 0.05, ^**^P < 0.01, ^***^P < 0.001. C3 propionate, C4 butyrate, IL-1β interleukin 1 beta, RANKL receptor activator of NF-κB ligand, TRAP tartrate resistant acid phosphatase, MMP9 matrix metalloprotein 9, CTSK cathepsin K

After confirming the suppressive effect of C3 and C4 on osteoclast differentiation, we continued to explore the roles of C3 and C4 in osteoclast lytic activity. Several genes such as *Trap*, carbonic anhydrase 2 (*Car2*), *Ctsk*, and *Mmp9* are crucial for bone resorption and extracellular matrix degradation. These related genes showed lower expression in the groups treated with C3 or C4 (*P* < 0.05, Fig. 6a). The detection of TRAP, CTSK, and MMP9 by Western blotting also confirmed this result (*P* < 0.001; Fig. 6b, c). TRAP staining and the immunohistochemical staining of NFATc-1, MMP9, and CTSK in calvaria slices were performed to verify the result of our in vitro study. It showed that C3 and C4 greatly reduced osteoclast activation as well as the number of NFATc-1-, CTSK-, and MMP9-positive cells (*P* < 0.001, Additional file 1: Fig. S7). Next, we investigated the effects of C3 and C4 on the podosome arrangement, a marker of osteoclast resorption. In osteoclasts differentiated with RANKL alone or with RANKL and IL-1β, F-actin was arranged in dense podosome belts and actin rings (the sealing zone), respectively, while osteoclasts treated with C3 or C4 showed scattered podosomes and failed to form morphologically normal podosome belts (Fig. 6d). Obviously, these findings confirmed that C3 and C4 affected the maturation and organization of osteoclast cytoskeletal structures required for bone resorption.

### Inhibitory effect of C3 and C4 on osteoclasts is mainly via suppression of histone deacetylase

**Fig. 7:**
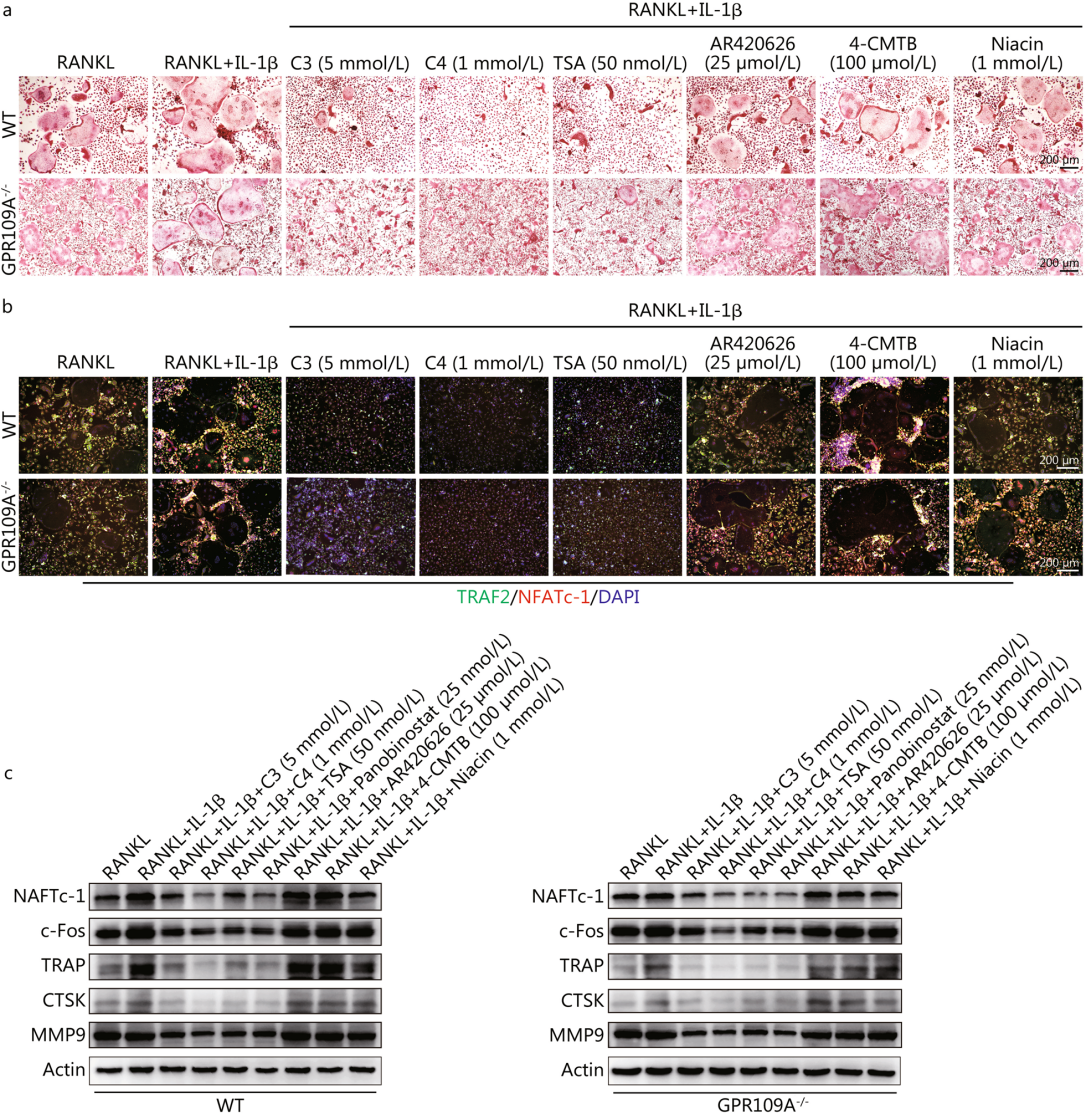
C3 and C4 affect osteoclast differentiation dependent on suppressing histone deacetylase. **a** Representative images of TRAP-positive multi-nucleated (≥ 5 nuclei) osteoclasts. **b** Representative immunofluorescence co-immunostaining images of osteoclasts stained with TRAF2 (green), NFATc-1 (red), and DAPI (blue). **c** Relative expression of osteoclast-related proteins. C3 propionate, C4 butyrate, IL-1β interleukin 1 beta, TSA trichostatin A, TRAP tartrate resistant acid phosphatase, NFATc-1 nuclear factor of activated T-cells, cytoplasmic 1, MMP9 matrix metalloprotein 9, CTSK cathepsin K

Like the potential mechanism of C3 and C4 on inhibition of inflammasome activation, whether C3 and C4 affect osteoclast differentiation through GPCRs or by suppressing HDAC activity remains unknown. To understand the role of HDACs and GPCRs in the effects of C3 and C4 on osteoclast differentiation stimulated by IL-1β, we used HDAC inhibitors (TSA, Panobinostat) and GPR41, GPR43, and GPR109A agonists (AR420626, 4-CMTB, niacin). We found that three GPCRs agonists did not affect osteoclast formation, while osteoclast differentiation was obviously suppressed by treatment with TSA (Fig. 7a and Additional file 1: Fig. S8). More importantly, the effect of TSA on osteoclast differentiation was not altered in GPR109A-deficient cells. Next, we analysed osteoclast differentiation-related proteins by Western blotting and immunofluorescence. These results also confirmed the negative effects of C3 and C4 in the differentiation of osteoclasts (Fig. 7b, c). Taken together, these results showed that the inhibitory effects of C3 and C4 on osteoclast differentiation were due to suppression of HDAC rather than activation of GPCRs.

## Discussion

In recent years, the specific mechanism underlying the osteolysis induced by wear particles has attracted much attention [44–49]. Increasing evidence has confirmed that activation of the NLRP3 inflammasome by wear particles is a key step in the course of osteolysis [24–27, 50]. Burton et al. [50] showed that caspase-1 deficiency markedly reduced osteolysis in vivo. Although the relationship between the inflammasome and osteolysis has been demonstrated, the underlying mechanism has never been clarified. In previous studies, it was found that wear particles stimulated IL-1β release and caspase-1 (pro) expression in vitro [50]. However, no studies have detected the cleavage of caspase-1 and IL-1β, a hallmark of NLRP3 inflammasome activation, in the supernatant except our previous work [28]. As activated caspase-1 leads to the cleavage of GSDMD followed by pyroptosis, the expression of cleaved caspase-1 should be analysed in the supernatant [40]. However, no work has investigated whether the activation of caspase-1 by wear particles induced pyroptosis. In this study, we detected GSDMD-NT, an effector protein in pyroptosis, and found that the NLRP3 inflammasome activation induced by wear particles also caused cell pyroptosis like other Nlrp3 activators [40].

As discussed above, any treatment that can inhibit activation of the NLRP3 inflammasome will be a candidate for osteolysis therapy. SCFAs are well known for their anti-inflammatory and immunomodulatory effects, especially in relation to the NLRP3 inflammasome. For example, Yuan et al. [37] reported differential effects of SCFAs on endothelial cell inflammasome activation. The same anti-inflammatory effect of butyrate has also been confirmed to alleviate acute pancreatitis by inhibiting activation of the pancreatic and colonic NLRP3 inflammasome [51]. More recently, it was found that butyrate suppressed monosodium urate crystal-induced IL-1β secretion by suppressing class I histone deacetylases in vitro [52]. These findings suggested that identification of the effect of SCFAs on NLRP3 inflammasome deactivation may provide insights into the control of related diseases, such as wear particle-induced osteolysis. Although our previous study confirmed that butyrate alleviated osteolysis by inhibiting NLRP3 inflammasome activation in BMDMs when exposed to wear particles, the upstream mechanism of inflammasome activation is still unknown. Furthermore, the roles of C2 and C3 in osteolysis have also not been investigated. In this study, we demonstrated that C3 and C4 rather than C2 do have an obvious therapeutic effect in the course of osteolysis in vivo. Next, we showed that C3 and C4 prevented CoCrMo alloy particle-induced ASC oligomerization and speck formation to control NLRP3 inflammasome activation, while C2 was not able to deactivate the inflammasome activation. Although Xu et al. [53] have shown that C2 effectively suppresses the activation of the NLRP3 inflammasome triggered by an Nlrp3 activator such as adenosine 5′-triphosphate (ATP), the same inhibitory effect of C2 on wear particle-induced inflammasome activation could not be observed in our study. Furthermore, we also discussed the underlying mechanism via which C3 and C4 inhibit inflammasome activation by activating the corresponding G protein-coupled receptor or acting as a histone deacetylase (HDAC) inhibitor. Our in vitro study revealed that C4 required the GRP109a receptor to restrict the NLRP3 inflammasome activation, while C3 did not depend on GPCRs or HDAC inhibition. Although niacin, a GPR109A agonist, had almost no inhibitory effect on activation of the inflammasome at a concentration of 100 μmol/L in another study, the benefits of C4 significantly diminished after GPR109A receptor knockout. In the present study, the inhibitory effect of niacin on inflammasome activation could be observed when the concentration of reached 1 mmol/L. These different results may be attributed to the concentration of niacin, our work showed that the benefits of C4 mainly depend on the GPR109A receptor. Furthermore, the specific mechanism of C3 on inflammasome activation may be more similar to that observed in previous studies into the effect of β-hydroxybutyrate on the inflammasome, which is independent of GPCRs or HDAC suppression [54].

Osteoclasts, as key players in osteolysis-related bone resorption, have been reported to be stimulated by inflammatory cytokines, especially IL-1β [25, 55–58]. Given that IL-1β is the main product of inflammasome activation and pyroptosis, we also investigated the effects of C3 and C4 on osteoclast differentiation stimulated by IL-1β. Surprisingly, C3 and C4 not only suppressed the secretion of IL-1β but also had the same effect on IL-1β-induced osteoclast differentiation, which is similar to that found in previous studies on the inhibition of osteoclasts by SCFAs [33]. However, in contrast to previous studies, we found that C3 and C4 not only inhibited the differentiation of osteoclasts but also affected podosome arrangements, which in turn reduced the formation of dense podosome belts or actin rings (the sealing zone) and thus impaired the bone-resorbing ability of osteoclasts. Additionally, unlike the underlying mechanism of C3 and C4 on the NLRP3 inflammasome, we found that GPCRs were not required for C3 and C4 to affect osteoclast formation. Instead, they may achieve their effects by suppressing HDAC, because non-selective HDAC inhibitors significantly inhibited the differentiation of osteoclasts.

The benefits of SCFAs in regulating multiple diseases have been gradually revealed with the studies of metabolites of gut microbiota [30]. The application of SCFAs in osteoporosis, rheumatoid arthritis, and other bone loss pathologies has achieved considerable success[34, 36, 59], whereas few studies have focused on the effects of SCFAs on osteolysis. In the present study, we confirmed that only C3 and C4 rather than C2 alleviated the development of osteolysis. Furthermore, with the study of SCFAs in the treatment of inflammatory osteolysis, the upstream mechanism of inflammasome activation induced by wear particles has also been elucidated for the first time. More importantly, in addition to regulating inflammation, C3 and C4 also suppressed the abnormal activation of osteoclasts promoted by IL-1β and impaired the ability of bone resorption of osteoclast. Previous studies on the treatment of osteolysis mostly focused on inhibiting inflammation or osteoclast activation, which was accompanied by obvious side effects [60].

Despite the therapeutic effects of C3 and C4 have been elucidated, there are still some limitations in the present study: (1) A human macrophage cell line (PMA-differentiated THP-1 macrophages) is not sufficient to validate the inhibition of inflammasome activation by C3 and C4 in human macrophages. Human primary macrophages need to be applied to verify the finding. (2) Although the exact mechanism was not investigated in this study, the different intervening concentration of niacin and other agonists should be applied to confirm the role of GPR109A receptor during the NLRP3 inflammasome activation. (3) As a part of bone metabolism, osteoblast could repair bone defects caused by osteolysis. Whether C3 and C4 partially alleviated osteolysis by promoting the differentiation or/and function of osteoblasts needs further investigation.

As a beneficial metabolite of gut microbiota, SCFAs could simply be produced by supplementation with a high fibre diet, which means that the intervention would be available immediately in the early stages of osteolysis without any side effects. The timely intervention for osteolysis at an early stage is very meaningful since patients at this point are often asymptomatic or undiagnosed [61]. If effective treatments are implemented in a timely manner, this would greatly reduce the possibility of developing aseptic loosening or even implant failure. However, without a definite diagnosis, it is unrealistic to administer drugs with side effects. Therefore, the revelation of the benefits of C3 and C4 on osteolysis will undoubtedly provide a qualified treatment with fewer side effects for those who may suffer from revision surgery.

## Conclusions

**Fig. 8:**
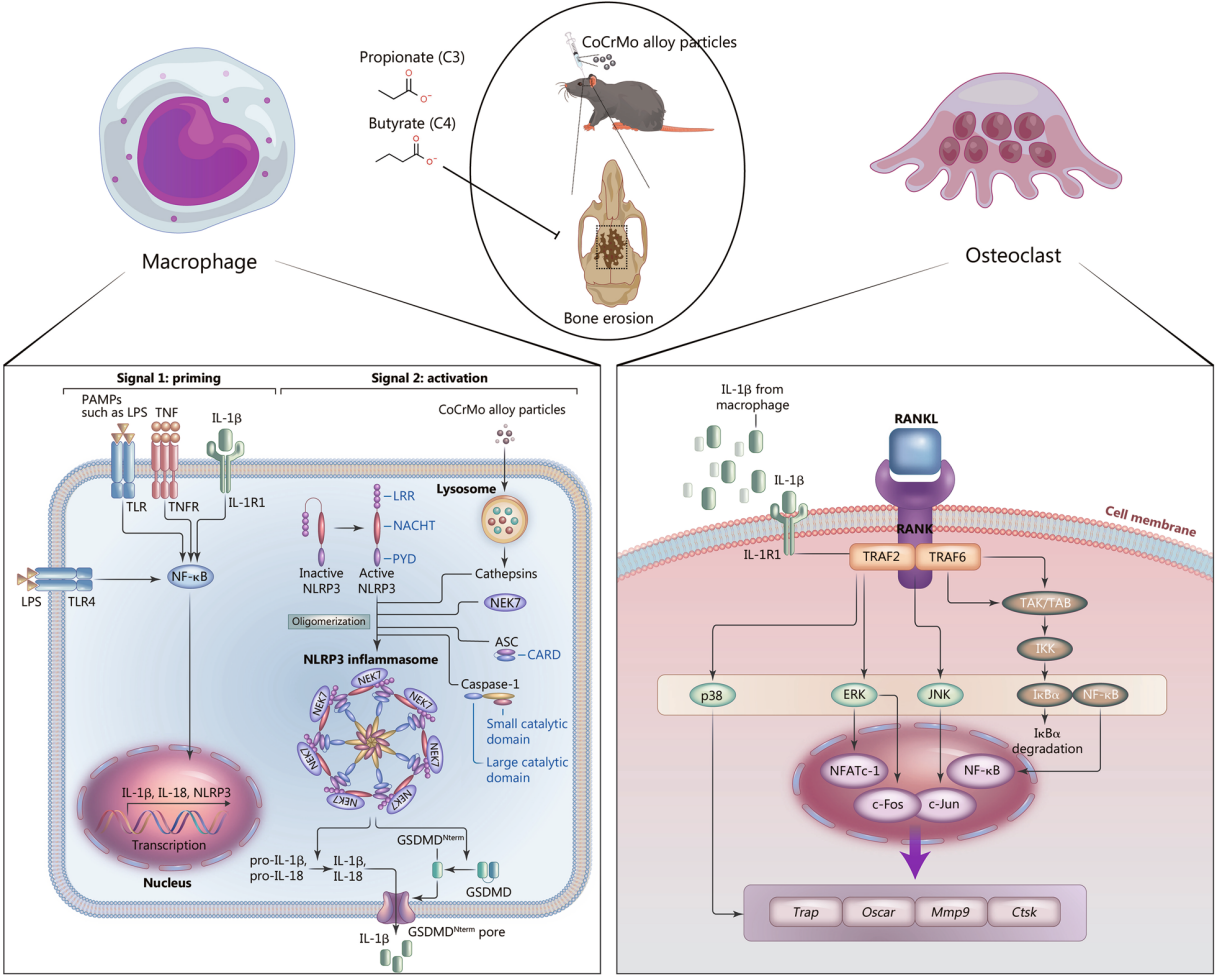
C3 and C4 alleviate osteolysis by inhibiting pyroptosis of macrophages and osteoclastogenesis

In summary, our work showed differential effects of SCFAs in the course of osteolysis: inhibition of pyroptosis following NLRP3 inflammasome activation and osteoclast formation stimulated by IL-1β (Fig. 8). As natural metabolites of gut microbiota, propionate and butyrate are undoubtedly effective treatments for preventing osteolysis induced by wear particles.

## Supplementary Information

**Additional file 1: Fig. S1** Purity of bone marrow derived macrophages (BMDMs). **Fig. S2** C3 and C4 inhibit NLPR3 inflammasome activated by CoCrMo alloy particles in macrophages. **Fig. S3** C3 and C4 suppressed the pyroptosis induced by CoCrMo alloy particles. **Fig. S4** C3 and C4 suppressed the pyroptosis in BMDMs. **Fig. S5** C3 inhibits the NLRP3 inflammasome activation independently of GPCRs and HADC inhibitor, while C4 is dependent on GPR109A receptor. **Fig. S6** Quantification of osteoclast-related proteins. **Fig. S7** C3 and C4 inhibit osteoclast differentiation and formation in vivo. **Fig. S8** Quantification of TRAP-positive multi-nucleated (≥ 5 nucleus) osteoclasts.

**Additional file 2: Table S1** Primer sequences in qRT-PCR analysis

## Data Availability

The datasets generated and/or analysed during the current study are not publicly available but are available from the corresponding author on reasonable request.

## Abbreviations

ANOVA: Analysis of variance
ASC: Apoptosis-associated speck-like protein containing a caspase recruitment domain
ATP: Adenosine 5′-triphosphate
BMD: Bone mineral density
BMDMs: Bone marrow-derived macrophages
BV/TV: Bone volume to tissue volume
C2: Acetate
C3: Propionate
C4: Butyrate
CTSK: Cathepsin K
Car2: Carbonic anhydrase 2
DMEM: Dulbecco’s modified eagle’s medium
DAPI: 2-(4-Amidinophenyl)-6-indolecarbamidine dihydrochloride
GPCR: G protein-coupled receptor
GPR109A: G protein-coupled receptor 109A
GSDMD: Gasdermin D
GSDMD-NT: GSDMD-N-terminal fragment
HDAC: Histone deacetylase
HE: Haematoxylin and eosin
IL-1β: Interleukin 1 beta
IL-6: Interleukin 6
IL-18: Interleukin 18
LPS: Lipopolysaccharide
M-CSF: Macrophage colony stimulating factor
Micro-CT: Micro-computed tomography
MMP9: Matrix metalloprotein 9
MSU: Monosodium urate
NFATc-1: Nuclear factor of activated T-cells, cytoplasmic 1
NF-κB: Nuclear factor kappa-B
NLRP3: Nod-like receptor pyrin domain 3
Ocstamp: Osteoclast stimulatory transmembrane protein
Oscar: Osteoclast-associated immunoglobulin-like receptor
PGE-2: Prostaglandin E2
PMA: Phorbol-12-myristate-13-acetate
PVDF: Polyvinylidene fluoride
RANKL: Receptor activator of NF-κB ligand
RIPA: Radio Immunoprecipitation Assay
SCFA: Short chain fatty acids
SDS-PAGE: Sodium dodecyl sulphate polyacrylamide gel electrophoresis
SPF: Specific pathogen free
THP-1: Tohoku hospital pediatrics-1
TJR: Total joint replacements
TNF: Tumour necrosis factor
TRAF2: TNF receptor-associated factor 2
TRAF6: TNF receptor-associated factor 6
TRAP: Tartrate resistant acid phosphatase
TSA: Trichostatin A
WT: Wild type
