# Supplementary material for: Propionate and butyrate attenuate macrophage pyroptosis and osteoclastogenesis induced by CoCrMo alloy particles
Source: Mil Med Res. 2022 Aug 23;9:46. doi: 10.1186/s40779-022-00404-0 (PMC9396885; doi:10.1186/s40779-022-00404-0)
Supplement: Supplementary file 1 — Additional file 1: Fig. S1 Purity of bone marrow derived macrophages (BMDMs). Fig. S2 C3 and C4 inhibit NLPR3 inflammasome activated by CoCrMo alloy particles in macrophages. Fig. S3 C3 and C4 suppressed the pyroptosis induced by CoCrMo alloy particles. Fig. S4 C3 and C4 suppressed the pyroptosis in BMDMs. Fig. S5 C3 inhibits the NLRP3 inflammasome activation independently of GPCRs and HADC inhibitor, while C4 is dependent on GPR109A receptor. Fig. S6 Quantification of osteoclast-related proteins. Fig. S7 C3 and C4 inhibit osteoclast differentiation and formation in vivo. Fig. S8 Quantification of TRAP-positive multi-nucleated (≥ 5 nucleus) osteoclasts. [file 40779_2022_404_MOESM1_ESM.pdf]

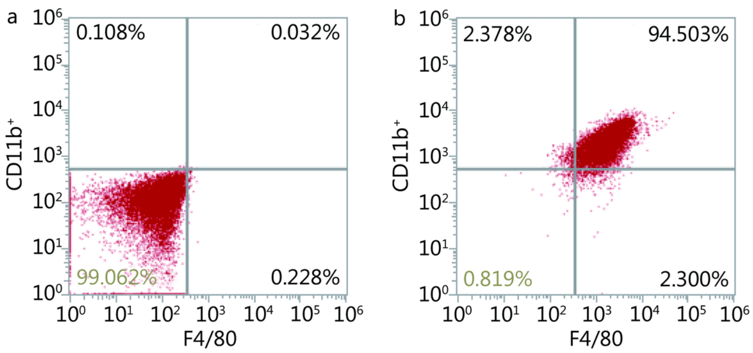

Fig. S1 Purity of bone marrow derived macrophages (BMDMs). **a** BMDMs without CD11b and F4/80 antibodies. **b** BMDMs with CD11b and F4/80 antibodies

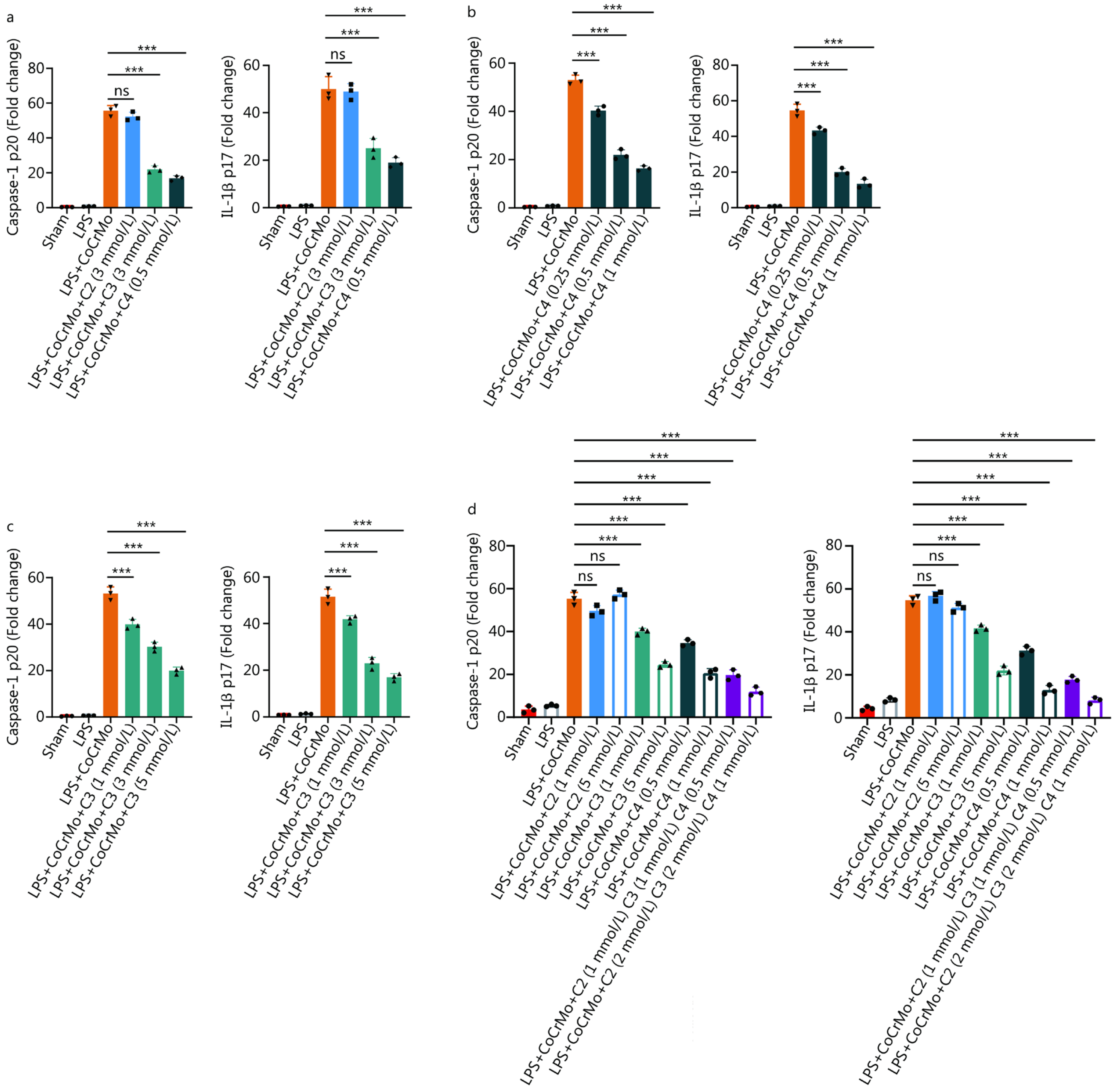

Fig. S2 C3 and C4 inhibit NLPR3 inflammasome activated by CoCrMo alloy particles in macrophages. BMDMs (LPS-primed) were treated with different doses of C2, C3, C4, and then stimulated with CoCrMo alloy particles. Cell lysates and supernatants were detected by immunoblotting. **a-c** Quantitation of caspase-1 p20 and IL-1β p17 band intensity as fold change. LPS-primed THP-1 macrophages (PMA-differentiated) treated with C2, C3, C4, and then stimulated with CoCrMo alloy particles. Supernatants were detected by immunoblotting. **d** Quantitation of caspase-1 p20 and IL-1β p17 band intensity as fold change. Results are mean ± SEM, $^{***}P < 0.001$, ns non-significant. C2 acetate, C3 propionate, C4 butyrate, LPS lipopolysaccharide, PMA phorbol-12-myristate-13-acetate

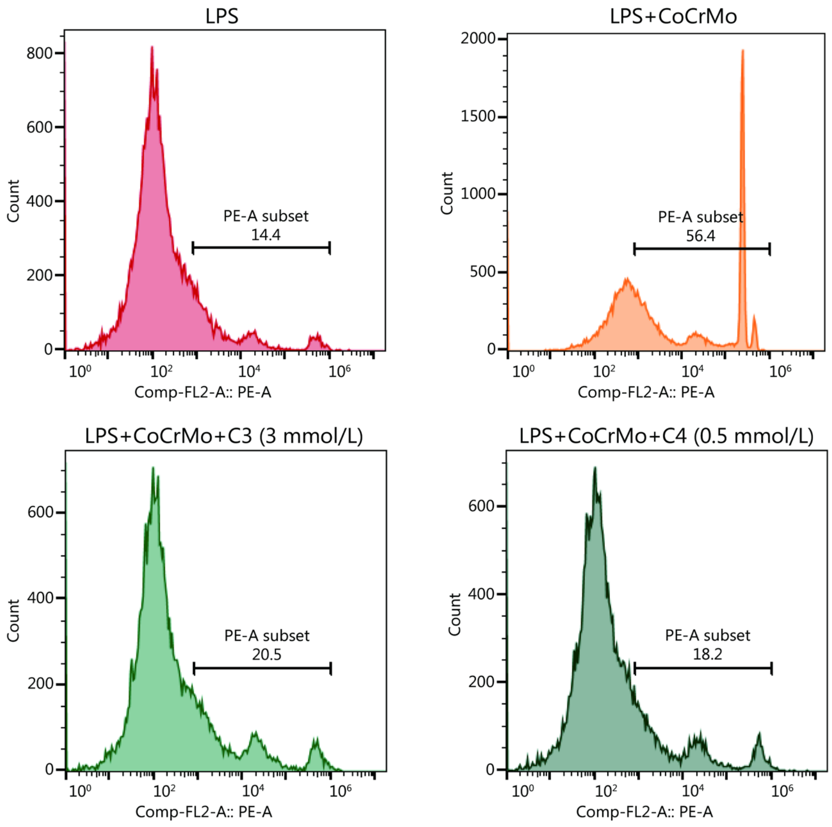

Fig. S3 C3 and C4 suppressed the pyroptosis induced by CoCrMo alloy particles. BMDMs (LPS-primed) were treated with C3 (3 mmol/L), C4 (0.5 mmol/L), and then stimulated with CoCrMo alloy particles. Cells were collected for PI staining and detected by flow cytometry. C3 propionate, C4 butyrate, BMDMs bone marrow derived macrophages, LPS lipopolysaccharide

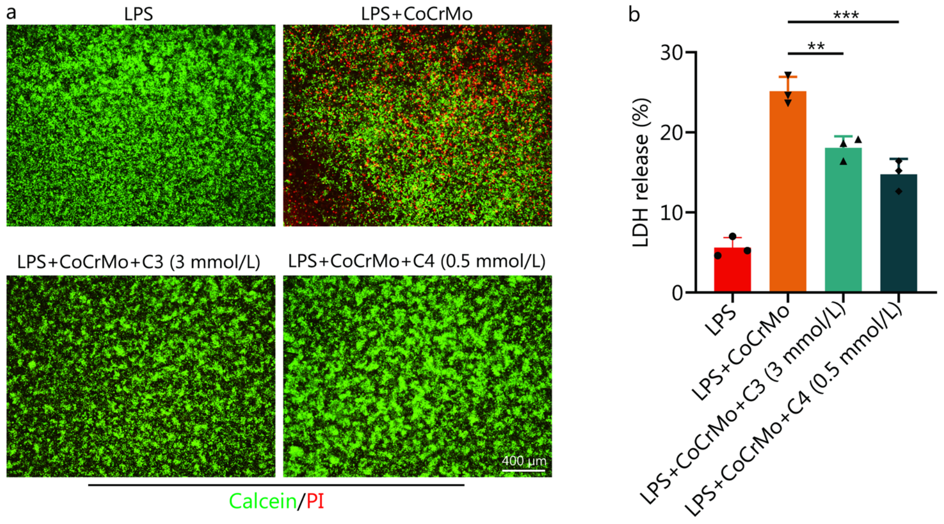

Fig. S4 C3 and C4 suppressed the pyroptosis in BMDMs. BMDMs (LPS-primed) were treated with C3 (3 mmol/L), C4 (0.5 mmol/L), and then stimulated with CoCrMo alloy particles. Cells were collected for immunofluorescence of Calcein/PI staining **(a)** and detection of LDH release **(b)**. Results are mean ± SEM, $^{**}P < 0.01$, $^{***}P < 0.001$. C3 propionate, C4 butyrate, BMDMs bone marrow derived macrophages, LPS lipopolysaccharide

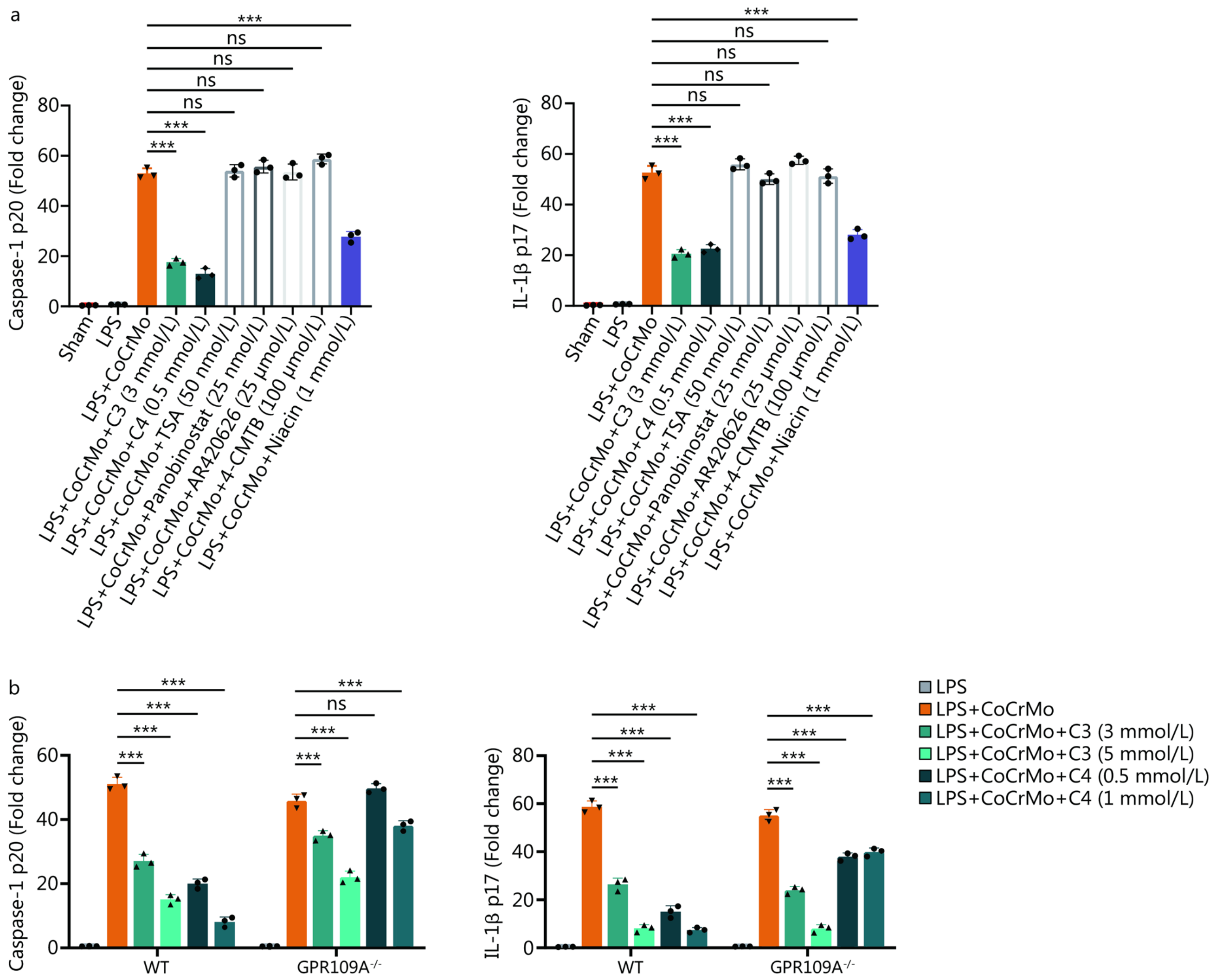

Fig. S5 C3 inhibits the NLRP3 inflammasome activation independently of GPCRs and HADC inhibitor, while C4 is dependent on GPR109A receptor. BMDMs (LPS-primed) treated with C3 (3 mmol/L), C4 (0.5 mmol/L), TSA (50 nmol/L), Panobinostat (25 nmol/L), AR42062 (25 μmol/L), 4-CMTB (100 μmol/L), Niacin (1 mmol/L) and then stimulated with CoCrMo alloy particles. **a** Supernatants were collected for immunoblotting. Quantitation of caspase-1 p20 and IL-1β p17 band intensity as fold change. BMDMs (LPS-primed) from wild type (WT) and GPR109A$^{-/-}$ mice treated with different doses of C3 or C4 and then stimulated with CoCrMo alloy particles. Supernatants were collected for immunoblottng. **b** Quantitation of caspase-1 p20 and IL-1β p17 band intensity as fold change. Results are mean ± SEM, $^{**}P < 0.01$, $^{***}P < 0.001$, ns non-significance. C3 propionate, C4 butyrate, BMDMs bone marrow derived macrophages, GPCR G protein-coupled receptor, GPR109A G protein-coupled receptor 109A, HADC histone deacetylase, IL-1β interleukin 1 beta, LPS lipopolysaccharide, NLRP3 Nod-like receptor pyrin domain 3, TSA trichostatin A

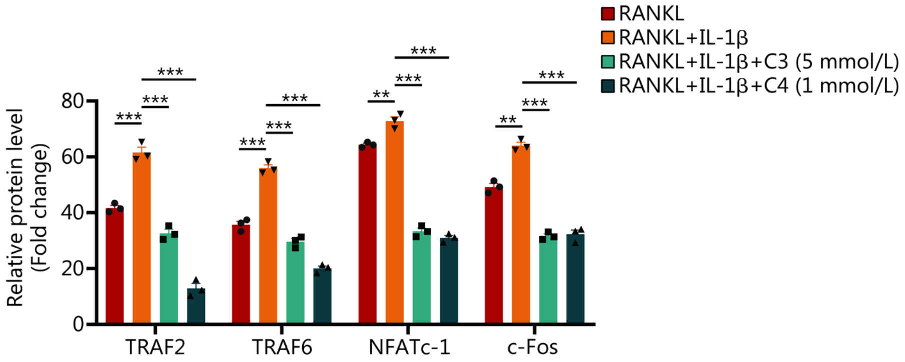

Fig. S6 Quantification of osteoclast-related proteins. Results are mean ± SEM, $^{**}P < 0.01$, $^{***}P < 0.001$. C3 propionate, C4 butyrate, RANKL receptor activator of NF-κB ligand, IL-1β interleukin 1 beta, TRAF2 TNF receptor-associated factor 2, TRAF6 TNF receptor-associated factor 6, NFATc-1 nuclear factor of activated T-cells, cytoplasmic 1

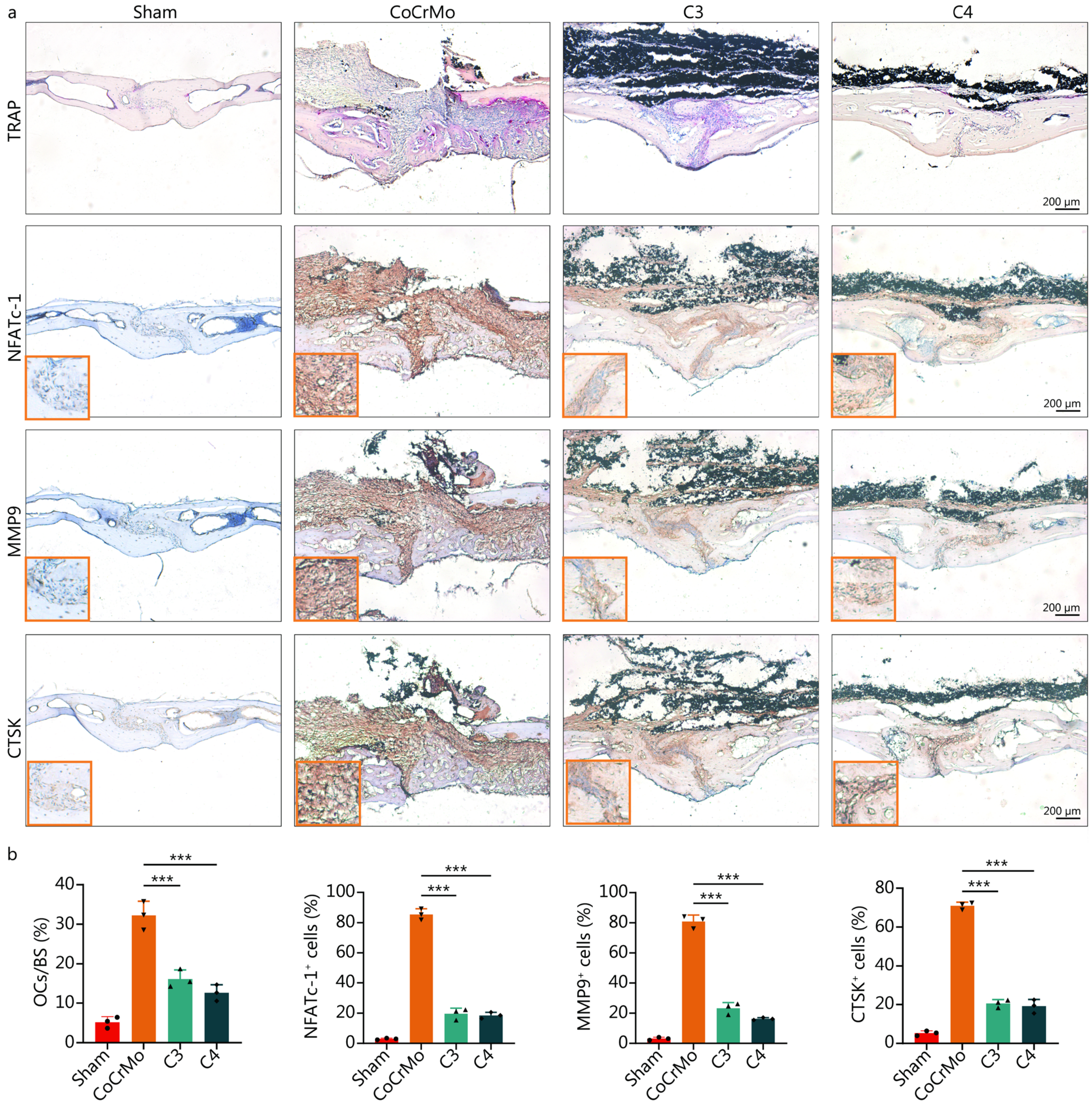

Fig. S7 C3 and C4 inhibit osteoclast differentiation and formation in vivo. **a** Representative TRAP staining of calvarial slices from each group. Representative immunohistochemical staining of NFATc-1, MMP9, and CTSK from each group. **b** Quantification of the percentage of osteoclasts surface per bone surface (OCs/BS, %), NFATc-1, MMP9, and CTSK positive cells. Results are mean ± SEM, $^{***}P < 0.001$. C3 propionate, C4 butyrate, TRAP tartrate resistant acid phosphatase, NFATc-1 nuclear factor of activated T-cells, cytoplasmic 1, MMP9 matrix metalloprotein 9, CTSK cathepsin K

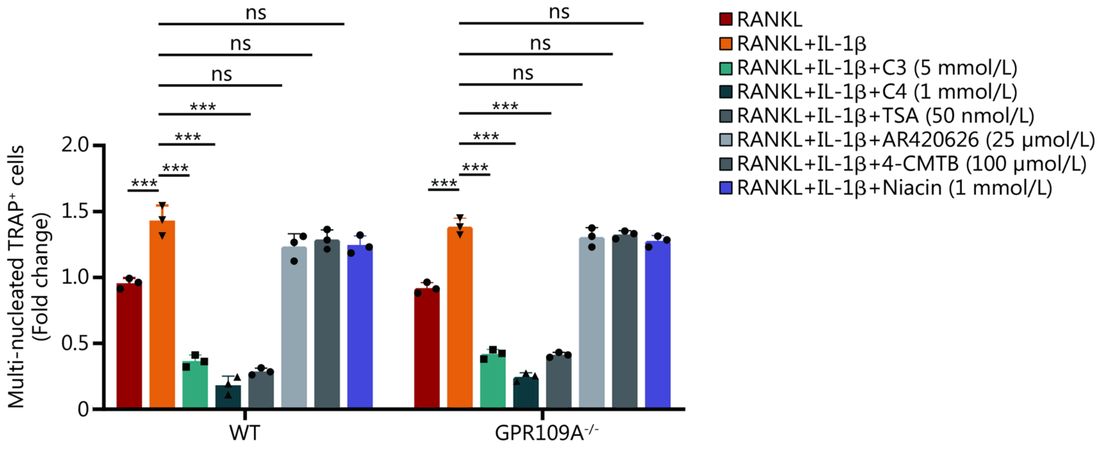

Fig. S8 Quantification of TRAP-positive multi-nucleated (≥ 5 nucleus) osteoclasts. Results are mean ± SEM, $^{***}P < 0.001$, ns non-significant. C3 propionate, C4 butyrate, RANKL receptor activator of NF-κB ligand, IL-1β interleukin 1 beta
