# Supplementary material for: Propionate and butyrate attenuate macrophage pyroptosis and osteoclastogenesis induced by CoCrMo alloy particles
Source: Mil Med Res. 2022 Aug 23;9:46. doi: 10.1186/s40779-022-00404-0 (PMC9396885; doi:10.1186/s40779-022-00404-0)
Supplement: Supplementary file 2 — Additional file 2: Table S1 Primer sequences in qRT-PCR analysis [file 40779_2022_404_MOESM2_ESM.pdf]

Table S1 Primer sequences in qRT-PCR analysis

Gene	Forward	Reverse
NFATc-1	5’-GGTGCCTTTTGCGAGCAGTATC-3’	5’-CGTATGGACCAGAATGTGACGG-3’
Ocstamp	5’-TTGCTCCTGTCCTACAGTGC-3’	5’-GCCCTCAGTAACACAGCTCA-3’
Oscar	5’-CTGCTGGTAACGGATCAGCTCCCCAGA-3’	5’-CCAAGGAGCCAGAACCTTCGAAACT-3’
Trap	5’-CGACCATTGTTAGCCACATACG-3’	5’-TCGTCCTGAAGATACTGCAGGTT-3’
Car2	5’-ACAGCAACTGCCCAGCAT-3’	5’-GAGCCCCAGTGAAAGTGAAA-3’
Ctsk	5’-AGGGCCAACTCAAGAAGAAAACT-3’	5’-TGCCATAGCCCACCACCAACACT-3’
Mmp9	5’-GCTGACTACGATAAGGACGGCA-3’	5’-TAGTGGTGCAGGCAGAGTAGGA-3’

qRT-PCR quantitative real-time polymerase chain reaction
